# Prohydrojasmon Promotes the Accumulation of Phenolic Compounds in Red Leaf Lettuce

**DOI:** 10.3390/plants10091920

**Published:** 2021-09-15

**Authors:** Shinya Takahashi, Yui Namioka, Haidar Rafid Azis, Tomoharu Sano, Mitsuko Aono, Masami Koshiyama, Hiroshi Fujisawa, Hiroko Isoda

**Affiliations:** 1 Faculty of Life and Environmental Sciences, University of Tsukuba, Tsukuba 305-8572, Japan; isoda.hiroko.ga@u.tsukuba.ac.jp; 2 Alliance for Research on the Mediterranean and North Africa (ARENA), University of Tsukuba, Tsukuba 305-8572, Japan; haidar.rafid.a@gmail.com; 3 Master’s Program in Life Science Innovation (T-LSI), University of Tsukuba, Tsukuba 305-8572, Japan; s1c111400591@gmail.com (Y.N.); maono@nies.go.jp (M.A.); 4 Health and Environmental Risk Division, National Institute for Environmental Studies, Tsukuba 305-8506, Japan; sanotomo@nies.go.jp; 5 Biodiversity Division, National Institute for Environmental Studies, Tsukuba 305-8506, Japan; 6 Specialty Chemicals Division, Zeon Corporation, Chiyoda, Tokyo 104-8246, Japan; M.Koshiyama@zeon.co.jp; 7 Headquarters, Zeon Corporation, Chiyoda, Tokyo 104-8246, Japan; cepi@zeon.co.jp

**Keywords:** anthocyanin, jasmonate, prohydrojasmon, phenolic acid, red leaf lettuce

## Abstract

Prohydrojasmon (PDJ) is a synthetic jasmonate derivative that is primarily used as a growth regulator, but its mechanism of action is unclear. In this study, we elucidated the effects of PDJ on phytochemical production in red leaf lettuce. The PDJ treatments promoted the accumulation of phenolic compounds in aerial plant parts. An LC-MS analysis revealed that these accumulated compounds were identified as cyanidin-3-*O*-glucoside, cyanidin-3-*O*-(6″-*O*-malonyl)-glucoside and cyanidin-3-*O*-(6″-*O*-malonyl)-glucoside methyl ester. The abundance of these compounds in lettuce extracts increased significantly in response to the PDJ treatment. Additionally, the LC-MS analysis also identified the accumulated phenolic compounds in the extracts of PDJ-treated lettuce, including caffeoyltartaric acid, chlorogenic acid, caffeoylmalic acid, chicoric acid, and dicaffeoylquinic acid. Gene expression analyses indicated the PDJ treatments upregulated the expression of *PAL*, *F3H*, and *ANS* genes in lettuce. These results suggest that PDJ treatments enhance the expression of genes involved in the synthesis of anthocyanins and phenolic compounds, resulting in an increase in the quantities of these compounds, which reportedly have various functions affecting human physiology.

## 1. Introduction

The demand for foods rich in phytochemicals has recently increased [1]. There is considerable epidemiological evidence that polyphenols promote health and decrease the risk of various diseases, including cancers and cardiovascular diseases [2,3]. Diverse vegetables and fruits are sources of polyphenols, and diets rich in polyphenols can help protect against several diseases [4,5].

Lettuce is a popular vegetable included in various foods to enhance human health. Its health-promoting properties are associated with its substantial abundance of antioxidants, primarily vitamin C and polyphenols, and fiber [6]. A previous study involving humans and rats revealed the protective effects of lettuce against cardiovascular diseases [7]. In red leaf lettuce (*Lactuca sativa* L.), quercetin, luteolin, cyanidin, chicoric acid, and caffeoylmalic acid are the major phytochemicals [8]. Phytochemical production in lettuce increases in response to environmental stresses, such as ultraviolet radiation, as well as injuries and postharvest processing [9].

Enhancing flavonoid biosynthesis pathways by modulating environmental conditions may be useful for promoting phytochemical production in crops. For example, cultivating lettuce plants in a greenhouse under low-temperature conditions reportedly induces the production of some types of phytochemicals [10]. Recent studies examined the utility of high-intensity artificial light, such as light-emitting diodes or ultraviolet light sources, for increasing lettuce anthocyanin contents [11,12]. Some genes encoding key enzymes in the phenylpropanoid pathway, including phenylalanine ammonia-lyase (PAL) and chalcone synthase (CHS), are light-inducible genes [13,14,15]. Additionally, some genes affecting anthocyanin synthesis pathways, including those encoding dihydroflavonol 4-reductase (DFR), leucoanthocyanidin dioxygenase (LDOX), and UDP-glucose: flavonoid 3-*O*-glucosyltransferase (UFGT), are jasmonic acid (JA)-inducible genes regulated by the phytochrome-mediated pathway in Arabidopsis [16].

Although altering light conditions is a convenient way to promote crop growth and phytochemical production during commercial crop production, installing new equipment and the associated power consumption may increase crop production costs.

Jasmonic acid and its derivatives are plant hormones and/or plant growth regulators in higher plants that control fruit ripening and coloration (including pigment accumulation). They can also induce the biosynthesis of secondary compounds, including phenylpropanoid derivatives, caffeic acids, flavonols, and anthocyanins [17,18]. Moreover, JA can improve crop quality by increasing secondary metabolite contents.

Prohydrojasmon [propyl (1*RS*,2*RS*)-(3-oxo-2-pentylcyclopentyl) acetate] (PDJ) is a synthetic JA analog. Earlier research proved that PDJ functions mimic those of JA in plants [19,20,21]. The use of PDJ as a plant growth regulator remains focused on fruits, including apples, oranges, mangoes, and grapes [20,22,23,24]. Specifically, it has been applied to enhance mango and red pear fruit coloration [25,26]. In our study, lettuce and komatsuna grown under artificial light were treated with several PDJ concentrations, which enhanced the production of some phenolics, polyphenols, and anthocyanins in lettuce leaves [27]. However, the precise mechanisms underlying PDJ-induced phenolic production in red leaf lettuce remain unclear.

In this study, we evaluated the effects of PDJ treatments on production of phenolic compounds in hydroponically grown red leaf lettuce. Red leaf lettuce (Lettuce) plants were cultivated in PDJ-supplemented culture media. We also quantitatively analyzed the components of methanolic extracts from PDJ-treated lettuce plants via colorimetric assays and high-performance liquid chromatography (HPLC). Furthermore, we investigated the compounds and molecular mechanisms in lettuce affected by PDJ using liquid chromatography–mass spectrometry (LC-MS)-based methods to identify the unknown compounds. We conducted gene expression analyses to clarify the regulation of enzymes involved in the phenylpropanoid derivative synthesis pathway in PDJ-treated lettuce.

## 2. Results

### 2.1. PDJ Treatments Increase the Phenolics Contents in Aerial Parts of Lettuce Plant

**Figure 1:**
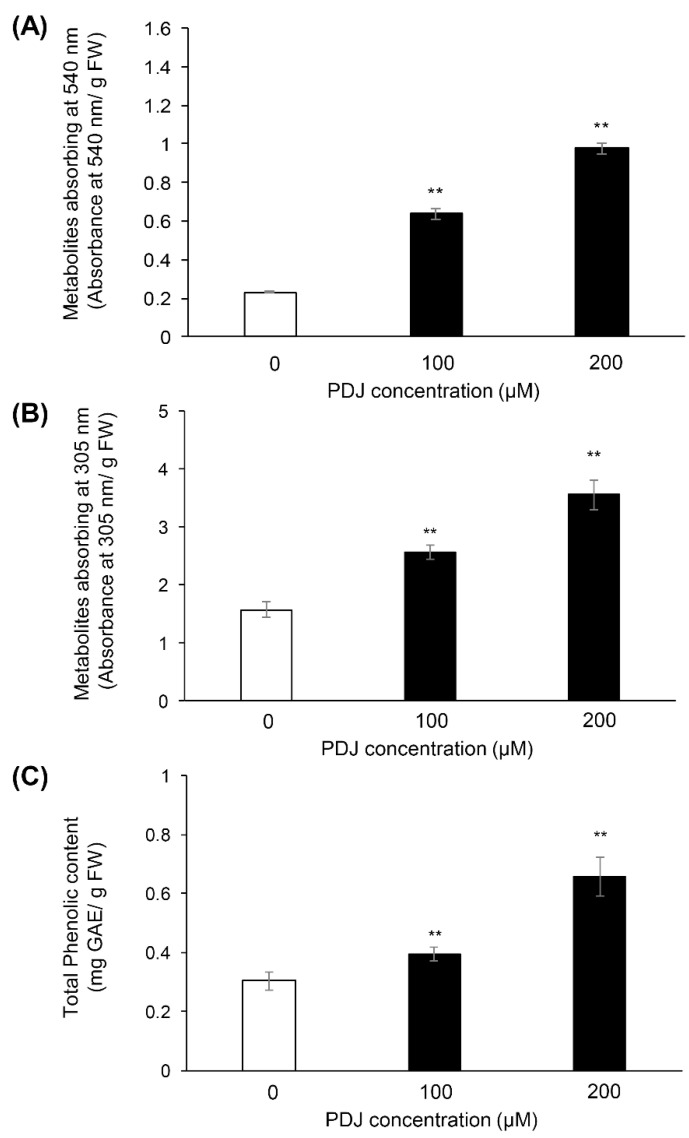
Amounts of compounds in extracts from aerial parts of PDJ-treated lettuce plant. Extracts from aerial parts of plants treated with (black) or without (white) 100 μM or 200 μM PDJ were measured the absorbance at 540 nm (**A**) and 305 nm (**B**). The extracts were reacted with phenolic reagents (**C**), for measurement of total phenolic content, measured the absorbance at 750 nm, respectively. Each value was calibrated with the fresh weight of the aerial parts. Each value indicated as mean ± standard error. Statistically significant differences are indicated by an asterisk (** *p* < 0.01).

Lettuce plants were grown hydroponically for 14 days after sowing and PDJ was supplied by irrigation for another 2 days. The first and second leaves were seen on the 16th day after sowing. The PDJ-treated lettuce showed red color compared to the lettuce without PDJ treatment (Supplementary Figure S1). We determined the contents of metabolites in the extracts from the aerial parts of lettuce plants treated with 0, 100 or 200 µM PDJ by measuring the absorbance at 540 and 305 nm. The metabolites absorbed at 540 nm containing the anthocyanin, were, respectively, 2.7-and 4.2-times higher in the lettuce plants treated with 100 and 200 µM than in the control plants (Figure 1A). The metabolites absorbed at 305 nm containing UV-light absorbing pigments (i.e., caffeic acid and flavonoid derivatives) were, respectively, 1.6- and 2.3-times higher in the lettuce plants treated with 100 and 200 µM than in the control plants (Figure 1B). We also determined the total phenolic content of the extracts from the aerial parts of lettuce plants treated with 0, 100 or 200 µM PDJ. Total phenolic content was 1.3-and 2.2-times greater in the lettuce plants treated with 100 and 200 µM PDJ than in the control plants, respectively (Figure 1C).

### 2.2. Extracts of PDJ-Treated Lettuce Plants Increased in Antioxidant Activities

**Figure 2:**
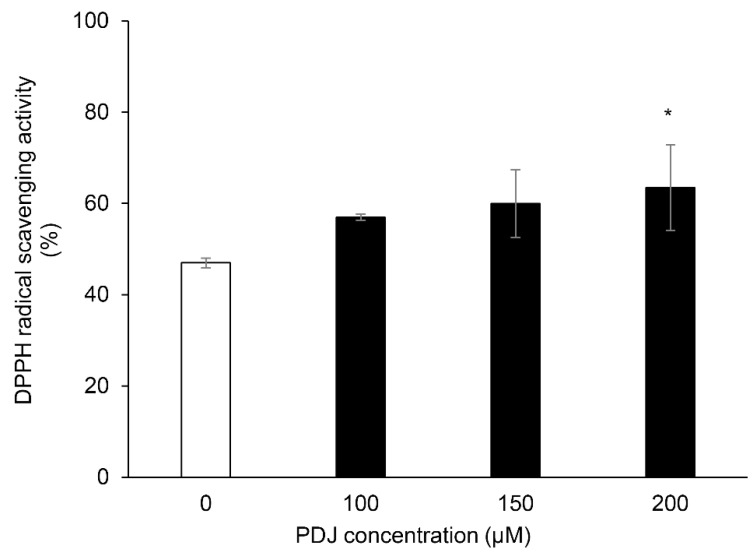
Radical scavenging activity in extracts from aerial parts of PDJ-treated lettuce plant. Extracts from aerial parts of plants treated with (black) or without (white) 100, 150, or 200 μM PDJ were reacted with DPPH reagents, for radical scavenging activity, and then measured the absorbance at 520 nm, respectively. Each value indicated as mean ± standard error. Statistically significant differences are indicated by an asterisk (* *p* < 0.05).

We conducted the DPPH radical scavenging assay to measure the antioxidant activities of the lettuce extracts. DPPH radical scavenging activities of the extracts from lettuce plants treated with 200 µM PDJ were higher than that of the control plants (Figure 2).

### 2.3. Quantification and Identification of the Pheolics in the Leaf Extracts from Lettuce Plants Treated with PDJ

**Figure 3:**
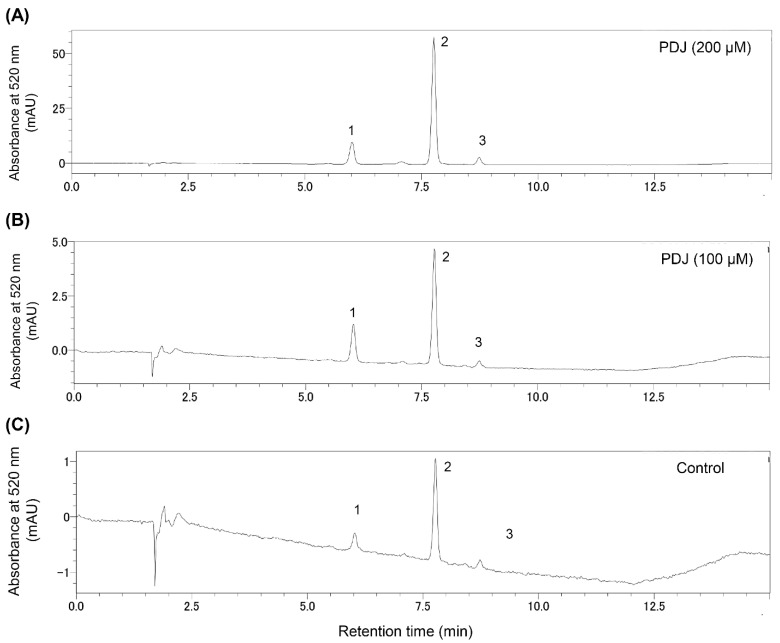
Chromatogram of compounds from the lettuce plant treated with PDJ obtained. Extracts from aerial parts of plants treated with 200 μM PDJ (**A**), 100 μM PDJ (**B**) or without (**C**) PDJ were analyzed by the HPLC method. Heights of the peak were indicated as absorbance at 520 nm (mAU). Peak1: cyanidin-3-O-glucoside (Cy3G), Peak2: cyanidin-3-O-(6″-O-malonyl)-glucoside (Cy3MG), and Peak3: cyanidin-3-*O*-(6″-*O*-malonyl)-glucoside methyl ester (Cy3MG-Me).

To clarify the changes to phenolics in PDJ-treated lettuce plants, we analyzed the extracts from the aerial parts of lettuce plants at 2 days after the PDJ treatment using an HPLC system. The chromatograms (at 520 nm) for the anthocyanins in the extracts from lettuce plants treated with 100 and 200 µM PDJ included three peaks (Figure 3).

The anthocyanins with increased contents induced by the PDJ treatments were analyzed by LC-MS. We determined all three peaks in Figure 3 from the LC-PDA-MS analysis data (Supplementary Figures S2–S6) comparing with the data of the literature. On the basis of an analysis of the mass spectra, the peaks at retention times of 5.97, 7.70, and 8.72 min were assigned to cyanidin-3-*O*-glucoside (Cy3G; M+, 449 *m*/*z*), cyanidin-3-*O*-(6″-*O*-malonyl)-glucoside (Cy3MG; M+, 535 *m*/*z*), and cyanidin-3-*O*-(6″-*O*-malonyl)-glucoside methyl ester (Cy3MG-Me; M+, 549 *m*/*z*), respectively, on the basis of the mass spectra, UV-vis spectra, and previous reports [28,29,30,31]. One of the three peaks was detected at the same retention time as the standard compound for cyanidin-3-*O*-glucoside using the HPLC system (Figure S7).

The Cy3G content of the PDJ-treated lettuce increased 2.5-and 6.39-times in responses to the 100 and 200 µM PDJ treatment, respectively. The Cy3MG content of the PDJ-treated lettuce increased 2.31- and 10.5-times in responses to the 100 and 200 µM PDJ treatment, respectively. The Cy3MG-Me content of the PDJ-treated lettuce increased 1.95-and 8.05-times in responses to the 100 and 200 µM PDJ treatment, respectively (Table 1 and Figure 3).

**Figure 4:**
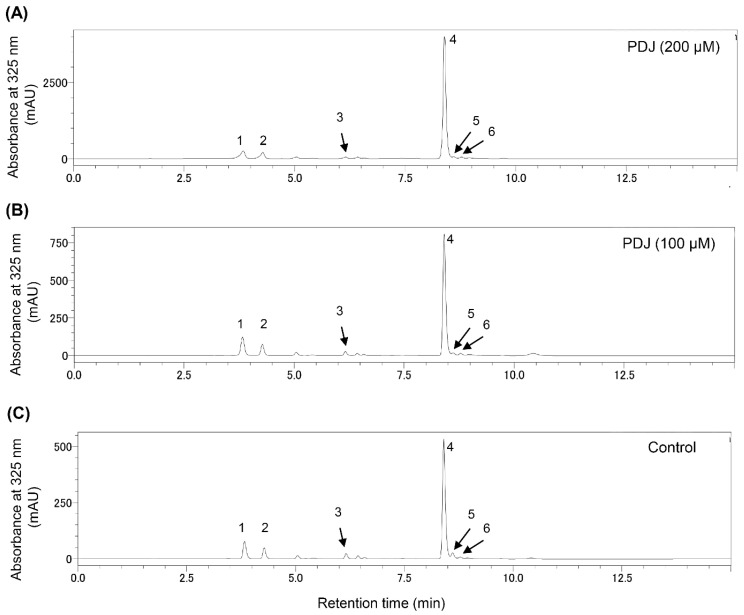
Chromatogram of compounds from the lettuce plant treated with PDJ obtained. Extracts from aerial parts of plants treated with 200 μM PDJ (**A**), 100 μM PDJ (**B**) or without (**C**) PDJ were analyzed by the HPLC method. Heights of the peak were indicated as absorbance at 325 nm (mAU). Peak1: caffeoyltartaric acid (CTA), Peak2: chlorogenic acid, Peak3: caffeoylmalic acid (CMA), Peak4: chicoric acid, Peak5: dicaffeoylquinic acid (diCQA), and Peak6: unidentified compound.

The chromatograms (at 325 nm) for the phenolics in the extracts from lettuce plants treated with 100 and 200 µM PDJ (Figure 4 and Table 2) comprised six peaks.

We determined all six peaks in Figure 4 from the LC-PDA-MS analysis data (Supplementary Figures S2–S6) comparing with the data of the literature [8,32,33,34,35,36,37]. Specifically, the peaks at retention times of 3.73, 4.41, 6.12, 8.34, 8.78, and 8.94 min were assigned to caffeoyltartaric acid (CTA; [M − H]^−^, 311 *m*/*z*), chlorogenic acid ([M − H]^−^, 353 *m*/*z*), caffeoylmalic acid (CMA; [M − H]^−^, 295 *m*/*z*), 2,3-dicaffeoyltartaric (chicoric) acid (2,3-diCTA; [M − H]^−^, 473 *m*/*z*), and dicaffeoylquinic acid (diCQA; [M − H]^−^, 515 *m*/*z*), respectively, on the basis of the mass spectra, UV spectra and previous reports [34,38,39]. It was suggested that peak 6 in Figure 4 may be the quercetin-3-*O*-(6″-*O*-malonyl)-glucoside (Q3MG; [M − H]^−^, 549 *m*/*z*) from the mass spectra and previous reports [34,38,39]. However, since no further evaluation data were available, we could not be identified as the Q3MG. Two of the six peaks were detected at the same retention times as each of the standard compounds for chlorogenic acid and chicoric acid using the HPLC system (Supplementary Figure S8).

The chicoric acid content increased 1.09- and 3.86-times in response to the 100 and 200 µM PDJ treatment, respectively. The content of CTA was 1.2- and 3.75 times higher in the extracts from lettuce plants treated with 100 and 200 µM PDJ, respectively, than in the control extract. The chlorogenic acid contents increased 1.17- and 4.56-times in response to the 100 and 200 µM PDJ treatment, respectively. The CMA contents increased 0.87- and 3.18-times in response to the 100 and 200 µM PDJ treatment, respectively. The diCQA contents increased 0.77-and 2.56-times in response to the 100 and 200 µM PDJ treatment, respectively (Table 2 and Figure 4).

### 2.4. Expression Analysis of Lettuce Genes Involved in the Phenylpropanoid Derivative Synthesis Pathway in PDJ-Treated Lettuce

We detected an increase in the contents of several anthocyanins and phenolic acids in PDJ-treated lettuce plants. In previous studies, some lettuce genes in the phenylpropanoid synthesis pathway were identified and investigated [11,40]. To clarify the mechanisms in lettuce affected by PDJ treatments, we analyzed the PDJ-induced gene expression changes in lettuce plants. We observed a slight difference in the degree of red color between the first and second leaf in the lettuce plantlets (Figure S1). Therefore, we determined the PDJ-induced gene expression changes in the first and second leaf of the lettuce aerial parts.

We selected the following six genes for a quantitative real-time PCR (qPCR) analysis: phenylalanine ammonia-lyase (*PAL*), chalcone synthase (*CHS*), flavanone 3-hydroxylase (*F3H*), flavonol synthase (*FLS*), dihydroflavonol 4-reductase (*DFR*), anthocyanidin synthase (*ANS*), and UDP-glucose: flavonoid 3-O-glucosyltransferase (*UFGT*). These genes encode key enzymes in the phenylpropanoid derivative synthesis pathway in lettuce [11].

**Figure 5:**
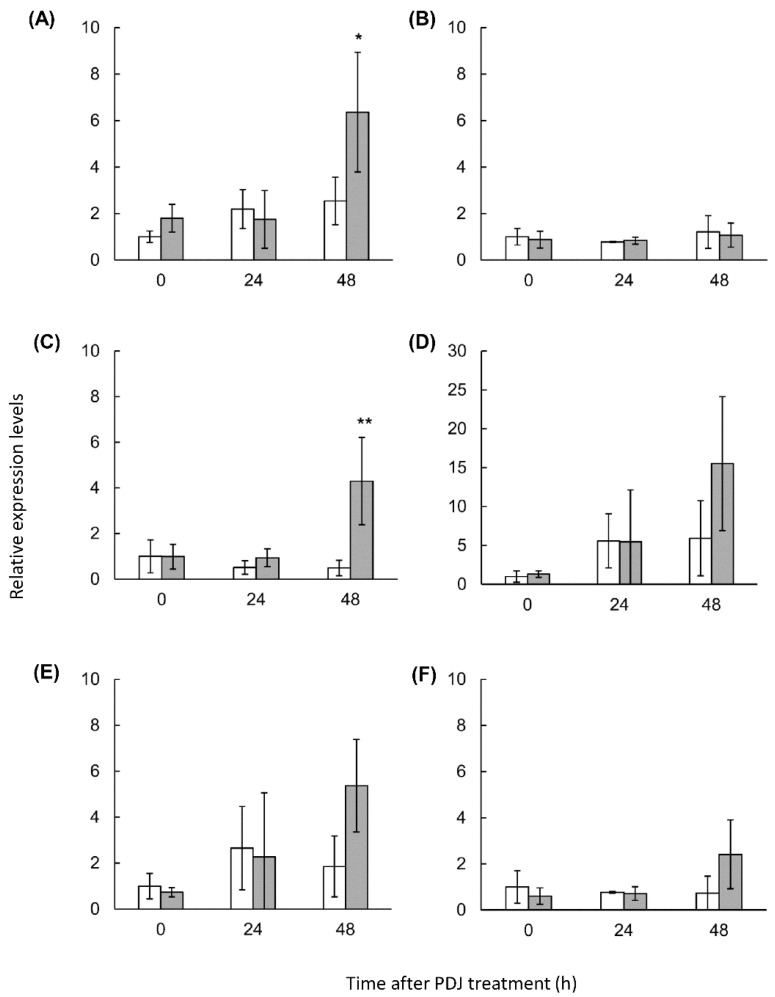
Gene expression of phenylpropanoid derivatives pathway in first leaves of lettuce plants treated with PDJ. The figure is 0 and 48 h after treatment. Gene-specific primer sets of *PAL* (**A**), *CHS* (**B**), *F3H* (**C**), *DFR* (**D**), *ANS* (**E**), and *UFGT* (**F**) were used to detect each gene expression by qPCR. Each value indicated as the average of relative expression three times using RNA samples obtained from three independent experiments. Statistically significant differences are indicated by an asterisk (* or ** indicate *p* < 0.05 or 0.01, respectively).

**Figure 6:**
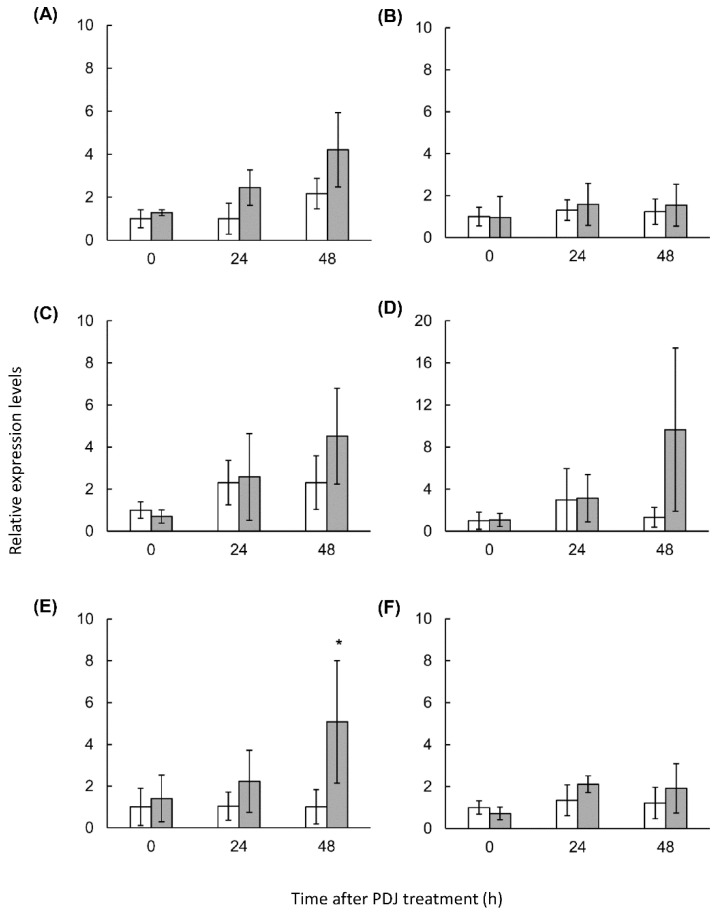
Gene expression of phenylpropanoid derivatives pathway in second leaves of lettuce plants treated with PDJ. The second leaves of the lettuce plant with (black) or without (white) PDJ were harvested at 0, 24, and 48 h after treatment. Gene-specific primer sets of *PAL* (**A**), *CHS* (**B**), *F3H* (**C**), *DFR* (**D**), *ANS* (**E**), and *UFGT* (**F**) were used to detect each gene expression by qPCR. Each value indicated as the average of relative expression three times using RNA samples obtained from three independent experiments. Statistically significant differences (*p* < 0.05) are indicated by an asterisk (*).

At 48 h after the PDJ treatment, the expression of most of the analyzed genes was upregulated in the first and second leaves of PDJ-treated lettuce plants. In the first leaf, the *PAL* (2.5-times), *F3H* (8.75-times), *DFR* (2.62-times), and *ANS* (2.89-times) expression levels were higher in the PDJ-treated lettuce plants than in the control plants, although the difference was significant only for *PAL* and *F3H*. Additionally, the *UFGT* expression level (3.29-times) also tended to be higher in PDJ-treated lettuce, relative to the control level (Figure 5). In the second leaf, the *PAL* (1.93-times), *F3H* (1.95-times), *DFR* (7.3-times), and *ANS* (5.04-times) genes tended to be more highly expressed in the PDJ-treated lettuce plants than in the control plants, but the difference was significant only for *ANS* (Figure 6). In contrast, there were no differences in the *CHS* expression levels between the PDJ-treated and control plants. These results suggested that the genes induced by the PDJ treatment influenced lettuce plant metabolism and promoted the production of caffeic acid derivatives and anthocyanin derivatives within 48 h.

## 3. Discussion

Prohydrojasmon, which is a synthetic JA derivative, has been used as a bio-stimulant to increase anthocyanin accumulation in crops [27]. Although PDJ is commercially available as a plant growth regulator useful for enhancing the coloration of various fruits, such as apple and grape, its mechanism of action has not been comprehensively characterized. Thus, in this study, we clarified its effects using leaves from hydroponically grown lettuce plants. In our preliminary experiments, we detected significant increases in the metabolites absorbing at 305 and 540 nm and the total phenolic content in the first and second leaf of lettuce plants at 1, and 2 days after a 200 μM PDJ treatment, especially in the first and second leaf at 2 days after the treatment (data not shown). Significant increases in the metabolites absorbing at 305 and 540 nm and the total phenolic content were detected in the lettuce plants treated with 100 μM PDJ, but they were lower than that of 200 μM PDJ treatment and no significant difference in the antioxidant activity at the 100 μM PDJ treatment. These differences between 100 and 200 μM PDJ may depend on the amounts of these compounds.

In this study, Cy3MG was the most abundant anthocyanin following the PDJ treatment. This anthocyanin is synthesized via the malonylation of Cy3G by anthocyanin 3-malonyl transferase [41].

We identified some malonylated compounds in the current study (e.g., Cy3MG). Malonylation enables the storage of phenolic compounds in vacuoles because it leads to increased water solubility and helps to stabilize the labile structure, which is conducive to the transport of compounds into the vacuole [42]. After entering cells, naphthol is mainly accumulated as a malonylglucoside, and some of it is released from cells in a methoxylated form and as a glycoside [42]. A treatment with PDJ may promote the malonylation of compounds, which are then transported and stored in vacuoles. Earlier investigations proved that Cy3MG can function as a superoxide scavenger [43] and that red leaf lettuce plants cultivated under cool conditions accumulate Cy3MG [44]. Because JA is a stress-inducible plant growth regulator, stress-induced responses may accelerate the accumulation of these malonylated compounds.

On the other hand, it was reported that the Cy3MG-Me was observed as a form of the methylated cyanidin malonylglucoside during elution of the anthocyanin with acidified MeOH [28,29]. In this process, Cy3MG reacted with the acidified MeOH and then esterification formed the methyl ester of Cy3MG. Additionally, finally, it converted to cyanidin-3-*O*-glucoside [29]. Since we used the acidified MeOH to extract the anthocyain, a part of Cy3MG might convert to the Cy3MG-Me and/or Cy3G.

CMA has the same *m*/*z* that of coutaric acid (CoTA). The retention time of the CoTA was 5.72 min. This was earlier than the retention time of 5-CQA (9.11 min) [38]. The retention time of CMA was later (13.04 min) than 5-CQA and CTA (3.75 min) [38]. In the present results, peak 3 in Figure 4 showed a later retention time than chlorogenic acid. Additionally, in the UV spectrum of this compound showed, the λmax was around 328 nm (Table 2 and Supplementary Figure S6), which was different from the λmax of CoTA (313 nm) [38]. Therefore, we determined that peak 3 in Figure 4 was the CMA.

The pathways involved in the synthesis of caffeic acid derivatives, such as chicoric acid, have not been thoroughly elucidated. However, a previous study on Arabidopsis confirmed that the pathway involved in the synthesis of caffeic acid derivatives diverges from *p*-coumaryl-CoA of the phenylpropanoid derivative synthesis pathway [45]. Phenylalanine ammonia-lyase is an early and key enzyme in the phenylpropanoid derivative synthesis pathway, and functions upstream of the synthesis of *p*-coumaryl-CoA. Methyl jasmonate treatments reportedly enhance PAL activity and increase the abundance of some phenolic compounds in radish [46]. We determined that *PAL* gene expression in lettuce is upregulated by PDJ treatments. A methyl jasmonate treatment can promote PAL activity and the expression of the *PAL* gene [47]. Exogenously applied PDJ can also increase the *PAL* gene expression level in red pear [26]. Thus, in lettuce plants, PDJ may enhance *PAL* expression, resulting in an increase in caffeic acid derivative contents. However, future studies regarding the regulated expression of other caffeic acid synthesis-related genes will need to clarify the PDJ-induced mechanisms.

We observed that the *PAL*, *F3H*, and *ANS* gene expression levels in PDJ-treated lettuce tend to increase after 48 h. In Arabidopsis, JA upregulates the expression of the late anthocyanin biosynthesis genes [48]. In the current study, we did not detect anthocyanin and flavonol intermediates, possibly because PDJ also induced the expression of the late anthocyanin biosynthesis genes (e.g., *DFR*, *ANS*, and *UFGT* genes), leading to the production of anthocyanin derivatives. Regarding the *ANS* and *DFR* genes, JA promotes anthocyanin accumulation in Arabidopsis plants through the COI1 (CORONATINE INSENSITIVE 1)–JAZ (JASMONATE ZIM-DOMAIN PROTEIN) transcriptional repression of the MYB75/PAP1 (R2R3 MYELOBLASTOSIS PROTEIN 75/PRODUCTION OF ANTHOCYANIN PIGMENT 1) transcription factors [48]. Furthermore, these transcription factors have a synergistic effect under far-red light conditions, which is mediated by the far-red light receptor phytochrome A (PHYA) [16]. A recent study indicated that endogenous JA is inactivated by a sulfotransferase (ST2a) in a process that is dependent on the PHYB-PIF pathway under low red:far-red light conditions [49]. In the present study, the artificial light sources used for our experiments emitted far-red light (700–780 nm). Thus, the transcriptional regulation of MYB through PHYA and COI1–JAZ as well as ST2a via the PHYB-PIF-mediated pathway may contribute to the anthocyanin accumulation in lettuce plants treated with PDJ.

**Figure 7:**
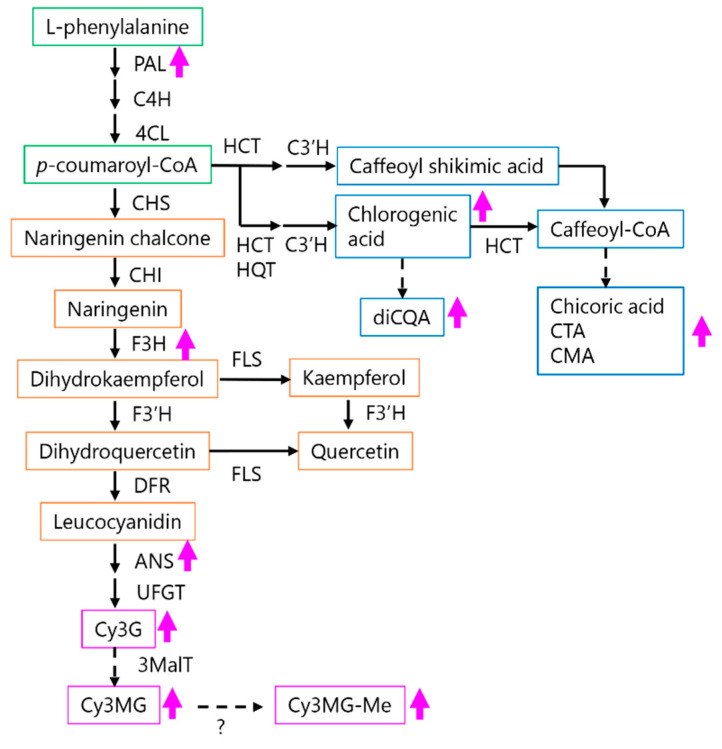
Predicted synthetic pathway of anthocyanin and phenolics in PDJ-treated lettuce plant. Increased amounts of compounds and expression of genes are indicated as arrows in magenta. Broken lines indicate unknown pathways. PAL: phenylalanine ammonia-lyase, CHS: chalcone synthase, CHI: chalcone isomerase, F3H: flavanone 3-hydroxylase, F3′H: flavonoid 3′-hydroxylase, FLS: flavonol synthase, DFR: dihydroflavonol-4-reductase, ANS: anthocyanidin synthase, and UFGT: UDP-glucose: flavonoid 3-O-glucosyltransferase, 3MalT: anthocyanin 3-malonyl transferase, MT: anthocyanin *O*-methyltransferase. HCT: hydroxycinnamoyl-CoA shikimate/quinate hydroxycinnamoil transferase, C3′H: p-coumaroyl ester 3′-hydroxylase, and HQT: hydroxycinnamoyl CoA quinate hydroxycinnamoyl transferase. The change from Cy3MG to Cy3MG-Me was presumed to be an artificial formation.

In our preliminary experiments, the total phenolic content and chicoric acid contents in lettuce plants grown under low-light conditions (photosynthetic photon flux density < 90 µE) did not increase following the PDJ treatment (data not shown). These results suggest that high-intensity light is required for the production of phenolic metabolites in lettuce plants. Moreover, an exposure to PDJ may enhance the production of these compounds via the upregulated expression of the corresponding genes (Figure 7). Becker et al. reported that the contents of some flavonoids, including Cy3G and Q3MG, increase in red leaf lettuce depending on the photosynthetic photon flux density, whereas phenolic acid contents, including chicoric acid, chlorogenic acid, and caffeoylmalic acid, are influenced by the leaf developmental stage rather than the photosynthetic photon flux density [44]. In future studies, we will investigate the relationship between light intensity and PDJ effects to determine the most appropriate way to improve phytochemical production in crops.

## 4. Materials and Methods

### 4.1. Plant Materials and Growth Conditions

Lettuce plants (*Lactuca sativa* L. cv. Red-fire) were grown under hydroponic conditions. Lettuce seeds were purchased from Takii and Co. Ltd. (Kyoto, Japan). The lettuce seeds were sown and germinated and the resulting seedlings were grown on Rockwool (Grodan, Roermond, The Netherlands) moistened with tap water in a growth chamber (HD-type Koitotron, Koito-Kogyo Co., Tokyo, Japan) at the National Institute for Environmental Studies, Japan. The temperature and relative humidity were maintained at 23 °C and 60% to 70%, respectively. Plants were exposed to a 14 h light/10-h dark cycle under fluorescent lights (National FPR96EX-N/A, Panasonic Co., Osaka, Japan) with a photosynthetic photon flux density of approximately 94 μmol m^−2^ s^−1^ at the top of each plant for 16 days.

### 4.2. PDJ Treatments

The lettuce seedlings were treated with a commercially available plant growth regulator, Jasmoneito Ekizai (Meiji Seika Pharma Co., Ltd., Tokyo, Japan), which contains 5% active PDJ (equivalent to 200 mM). Working solutions at specific concentrations (100, 150 and 200 μM) were prepared by dilution with tap water. Regarding the control treatment, tap water containing 30% (*v*/*v*) 1-propanol (Fujifilm-Wako Chemical Co., Osaka, Japan) and 0.03% (*v*/*v*) Tween 20 was used. At 14 days after sowing, PDJ and control solutions were supplied by irrigation only once under hydroponic conditions, and grown for another two days. For extraction of compounds absorbing at 305 and 540 nm and the total phenolic content, and for determination of antioxidant activity, aerial parts of lettuce plants (containing first and second leaves) were harvested 16 days after sowing. The aerial parts (fresh weight of each sample was about 0.13 g) of harvested lettuce were immediately frozen in liquid nitrogen and stored at −80 °C, after which they were lyophilized. In the aerial parts of the lettuce plant, water content was predicted about 92.7% calculated from fresh weight (just after harvested sample) and dry weight (lyophilized samples) (*n* = 18–20).

### 4.3. Extraction of Phenolics from Lettuce Leaves

Lyophilized lettuce samples (at 16 days after sowing) were ground to a powder and transferred to 15 mL tubes. The ground material was resuspended in a 10-fold volume of methanolic hydrochloric acid (80% methanol and 1% hydrochloric acid). The tubes were gently shaken to ensure the ground material was completely soaked. The tubes were incubated in darkness at 4 °C for 48 h. The obtained extracts were used for measuring amounts of the compounds absorbing at 305 and 540 nm and the total phenolic content.

### 4.4. Measurement of the Total Phenolic Contents of Lettuce Extracts

The total phenolic contents were measured using the Folin–Ciocalteu (FC) reagent as previously described [27]. The FC reagent was then added and the sample was incubated in darkness for 3 min. Next, 10% sodium carbonate (Tokyo Kasei Industry Co. Ltd., Tokyo, Japan) was added and the sample was incubated in darkness for 30 min. The absorbance (at 750 nm) of each extract per gram fresh weight of plant tissue was measured using a microreader (Varioskan LUX, Thermo Fisher Scientific Inc., Waltham, MA, USA). The total phenolic content, which was expressed as gallic acid equivalents (GAE), was calculated based on a standard curve generated using gallic acid solutions. The data are presented herein as milligrams of gallic acid equivalents per gram of plant tissue fresh weight (mg GAE/g FW). The analysis was conducted three times using samples obtained from three independent experiments.

### 4.5. Measurement of the Compounds Aabsorbing at 305 and 540 nm Contents of Lettuce Extracts

The compounds absorbing at 305 and 540 nm were measured as previously described, with some modifications [27]. Briefly, the absorbance at 305 nm and 540 nm of the methanolic hydrochloric acid extracts of plant tissues (whole aerial lettuce plant parts) were measured using the Varioskan LUX microreader. Each extract was transferred to a 96-well microplate. The compounds absorbing at 305 and 540 nm contents are herein expressed as the absorbance of the extract per gram of plant tissue fresh weight (Absorbance at 305 nm/g FW and Absorbance at 540 nm /g FW). The analysis was conducted three times using samples obtained from three independent experiments.

### 4.6. Measurement of the Antioxidant Activity of Lettuce Extracts

The antioxidant activity was measured using the DPPH (2,2-diphenyl-1-picrylhydrazyl) free radical scavenging assay as previously described [27,34]. Briefly, a DPPH working solution was prepared and Trolox was used to generate a standard curve. A 10 µL aliquot of the extract or Trolox was mixed with a 190 μL DPPH working solution. The sample was added to a microplate, which was incubated in darkness for 10 min at ambient temperature to allow the reaction to proceed. The absorbance at 520 nm was measured in triplicate for each DPPH (+) and DPPH (−) sample using the Varioskan LUX microreader. The DPPH radical scavenging activity was expressed as the percentage absorbance inhibition (%) calculated according to the following:

DPPH radical scavenging activity (%) = [(AB − AS)/AB] × 100

when AB: Absorbance of the blank; AS: Absorbance of the sample. The analysis was conducted three times using samples obtained from three independent experiments.

### 4.7. HPLC and LC-MS Analyses of the Extracts from PDJ-Treated Lettuce

The methanolic hydrochloric acid extracts were passed through filter paper (ADVANTEC, Tokyo, Japan), after which the filtrates were heated in a water bath at 40 °C and then dried using a rotary evaporator (EYELA Tokyo Rikakikai Co. Ltd., Tokyo, Japan). The volume was adjusted with methanolic hydrochloric acid for a sample concentration of 5 mg/mL. Extracts were stored at −30 °C until analyzed. The extracts were filtered using a 0.45 μm Syringe Driven Filter (Milex-LH, Merck Millipore Ltd., Tullagreen, Ireland) for the HPLC analysis, which was completed using the Ultimate 3000 system (Thermo Fisher Scientific, Waltham, MA, USA). More specifically, the extracts were separated and analyzed using the Acclaim C30 stationary phase column (pore size 3 µm, 3.0 mm × 150 mm; Thermo Fisher Scientific, Waltham, MA, USA). The mobile phases were 0.1% (*v*/*v*) trifluoroacetic acid (Fujifilm-Wako Chemical, Richmond, VA, USA) as solvent A and 100% acetonitrile (Fujifilm-Wako Chemical) as solvent B. The initial gradient conditions were 90% A and 10% B. In 10 min, the gradient reached 75% A and 25% B. In the following 10 min, the gradient was adjusted to 90% A and 10% B. The sample injection volume was 1 µL. The column flow rate was 0.5 mL/min at 30 °C. The absorbance was measured at 520 and 325 nm for anthocyanin and phenolic acids (hydroxycinnamic acids), respectively, using a diode array detector system (DAD-3000, Thermo Fisher Scientific, Waltham, MA, USA). As standard compounds, cyanidin-3-*O*-glucoside chloride (Nagara Science Co. Ltd., Gifu, Japan; purity on HPLC ≥ 99%), chlorogenic acid (Fujifilm-Wako Chemical Co., Osaka, Japan), and chicoric acid (Tokyo Chemical Industry Co. Ltd., Tokyo, Japan; purity on ≥ 98%). The cyanidin-3-*O*-glucoside chloride was diluted in distilled water. The chlorogenic acid and the chicoric acid were diluted in methanol. To quantify the amounts of anthocyanins and phenolic compounds, we made the standard curve of cyanidin-3-*O*-glucoside and chicoric acid. To construct the calibration curves, appropriate 10 mg/mL of cyanidin-3-*O*-glucoside chloride and chicoric acid were diluted with water and methanol, respectively. For cyanidin-3-*O*-glucoside chloride, four concentration levels (0.1, 0.15, 0.2 and 0.25 mg/mL) were analyzed. For chicoric acid, five concentration levels (0.05, 0.1, 0.15, 0.2 and 0.25 mg/mL) were analyzed. The calibration curve showed linearly, with correlation coefficients 0.9979 and 0.9863 for cyanidin-3-O-glucoside chloride and chicoric acid, respectively. For quantification, peak areas were correlated with concentrations in accordance with the calibration curves. For Cy3G, Cy3MG, and Cy3MG-Me, cyanidin-3-O-glucoside chloride was used for calibration. For CTA, chlorogenic acid, CMA, chicoric acid, diCQA, chicoric acid equivalent was used.

The amounts of compounds were expressed μg Cy3G equivalent (for anthocyain) or mg chicoric acid equivalent (for phenolic acids or flavonol derivative) per gram of plant material fresh weight.

The HPLC analysis was conducted three times using samples obtained from three independent experiments.

The LC-MS analyses were performed using the LCMS-8040 mass spectrometer with the Nexera X2 UHPLC system and the SPD-M30A diode array detector (Shimadzu Co., Kyoto, Japan). The LC conditions were the same as those used for the HPLC analysis, whereas the MS conditions were as follows: ionization mode: positive (for anthocyanins) and negative (for phenolics) electrospray ionization; capillary voltage: 4.5 kV; drying gas: 5 L/min; nebulizer gas: 2 L/min; desolvation line temperature: 250 °C; heat block temperature: 450 °C; and detection mode: selected ion monitoring (SIM). The *m*/*z* of the detected ions were 449 (Cy3G), 535 (Cy3MG), 549 (Cy3MG-Me), 311 (CTA), 353 (chlorogenic acid), 295 (CMA), 473 (chicoric acids), 515 (diCQA), and 549 (unidentified compound). The LC-MS analysis was conducted three times using samples obtained from the independent experiments.

### 4.8. Gene Expression Analysis of Phenylpropanoid Derivative Synthesis Genes in PDJ-Treated Lettuce

We analyzed the expression-level changes to the phenylpropanoid synthesis genes in PDJ-treated lettuce by conducting a qPCR assay. The first and second leaves of five lettuce plants treated with or without 200 μM PDJ were harvested at 0, 24 and 48 h after the treatments. The collected leaves were stored in RNAlater solution (Thermo Fisher Scientific, Waltham, MA, USA) at −20 °C until used. Total RNA was extracted from the harvested leaves using the RNAprep Pure Plant Plus Kit with RNase-Free DNase I (TIANGEN Biotech Co. Ltd., Beijing, China). Specifically, leaves in RNAlater solution were transferred to 1.5 mL microtubes, which were weighed before the leaves were ground using a pestle homogenizer. The RNA was extracted according to the manufacturer’s instructions. The total RNA quantity was determined using the NanoDrop 2000 spectrophotometer (Thermo Fisher Scientific, Waltham, MA, USA).

The extracted RNA served as the template for a reverse transcription using the Superscript IV VILO Master Mix kit (Thermo Fisher Scientific, Waltham, MA, USA). The total RNA (1 μg) was mixed with the DNase solution comprising 1 µL 10× ezDNase Buffer, 1 µL ezDNase enzyme, and nuclease-free water (up to 10 µL) to digest genomic DNA at 37 °C for 2 min. The mixture was then placed on ice. After adding 4 µL SuperScript IV VILO Master mix and 6 µL nuclease-free water, the solution was incubated at 25 °C for 10 min and then at 50 °C for 10 min. To inactivate the enzyme, the mixture was incubated at 85 °C for 5 min. The synthesized cDNA quantity was measured, after which the cDNA was stored at −20 °C until used.

The qPCR assay was performed using the 7500 Fast Real-Time System (Thermo Fisher Scientific, Waltham, MA, USA). We analyzed the expression of genes encoding chalcone synthase (*CHS*), phenylalanine ammonia-lyase (*PAL*), flavanone 3-hydroxylase (*F3H*), anthocyanin synthase (*ANS*), UDP-glucose: flavonoid 3-O-glucosyltransferase (*UFGT*), dihydroflavonol 4-reductase (*DFR*), and actin (*ACT*). We designed gene-specific primers based on the information provided in a published article (Kitazaki et al. 2018) (Table S1). The primers were purchased from Integrated DNA Technologies Inc. (USA).

The qPCR was completed using the KOD SYBR qPCR kit (TOYOBO Co. Ltd., Osaka, Japan). The synthesized cDNA (500 ng in 5.6 µL) was mixed with 10 µL KOD SYBR qPCR Mix, 4 µL primer solution (forward and reverse primers, 0.2 µM each), and 0.4 µL 50× ROX reference dye. The PCR conditions were as follows: 50 °C for 2 min and 95 °C for 10 min; 45 cycles of 95 °C for 15 s and 60 °C for 60 s. Expression data were obtained from three independent experiments. The expression levels of all analyzed genes were normalized against the *ACT* expression level. The expression data were analyzed using the comparative Ct (ΔΔCt) method of the 7500 Fast Real-Time System software (version 2.0.5) (Thermo Fisher Scientific, Waltham, MA, USA). The qPCR assay was conducted three times using RNA samples obtained from three independent experiments.

### 4.9. Statistical Analysis

All data were analyzed using Microsoft Excel. Significant differences were estimated based on an ANOVA test (*p* < 0.05 or 0.01).

## 5. Conclusions

We identified the lettuce compounds affected by PDJ treatments. Additionally, we revealed that the expression of some lettuce genes related to the synthesis of anthocyanins and phenolics is upregulated by exogenous PDJ. We determined that PDJ treatments enhance the expression of genes encoding enzymes involved in phenylpropanoid synthesis pathways, thereby promoting the production of caffeic acid derivatives and anthocyanin derivatives in lettuce.

## Figures and Tables

**Table 1:** Contents of individual anthocyanin derivatives in lettuce leaves after PDJ treatments.

Compound	Rt (min)	λmax (nm)	M+ *m*/*z*	Content [µg Cy3G eq./g Fresh Weight] (Mean ± SD)		
				0 µM	100 µM	200 µM
Cy3G	5.97	273, 520	449	1.66 ± 1.54 ^c^	4.19 ± 0.66 ^b^	10.6 ± 0.2 ^a^
Cy3MG	7.70	277, 519	535	2.43 ± 0.04 ^c^	5.62 ± 0.02 ^b^	25.6 ± 0.4 ^a^
Cy3MG-Me	8.72	270, 523	549	0.64 ± 0.02 ^c^	1.25 ± 0.08 ^b^	5.15 ± 0.08 ^a^

Rt: retention time; Cy3G: cyanidin-3-*O*-glucoside; Cy3MG: cyanidin-3-*O*-(6″-*O*-malonyl)-glucoside; Cy3MG-Me: Cyanidin-3-*O*-(6″-*O*-malonyl)-glucoside methyl ester. Significant different letters (^a–c^) in the same row are indicated by different superscript letters (*p* < 0.05).

**Table 2:** Contents of caffeic acid derivatives in lettuce leaves after PDJ treatments.

Compound	Rt (min)	UV λmax (nm)	[M − H]^−^ *m*/*z*	Content [mg Chicoric Acid eq./g Fresh Weight] (Mean ± SD)		
				0 µM	100 µM	200 µM
CTA	3.73	329	311	0.114 ± 0.001 ^c^	0.137 ± 0.002 ^b^	0.428 ± 0.005 ^a^
Chlorogenic acid	4.41	326	353	0.059 ± 0.0003 ^c^	0.069 ± 0.001 ^b^	0.269 ± 0.006 ^a^
CMA	6.12	328	295	0.038 ± 0.001 ^c^	0.033 ± 0.0001 ^b^	0.121 ± 0.002 ^a^
Chicoric acid	8.34	329	473	0.731 ± 0.028 ^c^	0.795 ± 0.015 ^b^	2.824 ± 0.025 ^a^
diCQA	8.78	327	515	0.030 ± 0.001 ^c^	0.023 ± 0.001 ^b^	0.077 ± 0.001 ^a^

Rt: retention time; CTA: caffeoyltartaric acid; CMA: caffeoylmalic acid; diCQA: dicaffeoylquinic acid. Significant different letters (^a–c^) in the same row are indicated by different superscript letters (*p* < 0.05).

## Data Availability

All our data, tables and figures in this manuscript are original. We have added our data availability statement. The data presented in this study are available in the article and its supplementary material.

## Supplementary Materials

The following are available online at https://www.mdpi.com/article/10.3390/plants10091920/s1, Figure S1: Red leaf lettuce seedling treated without or with prohydrojasmon (PDJ), Figure S2: Mass spectrometry chromatograms (Selected Ion Monitoring: SIM) of the extracts from lettuce leaves not treated with PDJ, Figure S3. Mass spectrometry chromatograms (SIM) of the extracts from lettuce leaves treated with 100 μM PDJ, Figure S4: Mass spectrometry chromatograms (SIM) of the extracts from lettuce leaves after 200 μM PDJ treatments, Figure S5: Mass spectrometry spectra (Scan) of the extracts from lettuce leaves with 200 μM PDJ treatments, Figure S6: UV-Vis spectra of the peaks found in the extracts from lettuce leaves with 200 μM PDJ treatments, Figure S7: Chromatograms of the metabolites absorbing at 520 nm from lettuce plants treated with or without PDJ, Figure S8: Chromatograms of the metabolites absorbing at 325 nm from lettuce leaves with or without PDJ treatments, Table S1: Sequences of gene-specific qPCR primers.
