# Supplementary figures and images for: Prohydrojasmon Promotes the Accumulation of Phenolic Compounds in Red Leaf Lettuce
Source: Plants (Basel). 2021 Sep 15;10(9):1920. doi: 10.3390/plants10091920 (PMC8468872; doi:10.3390/plants10091920)

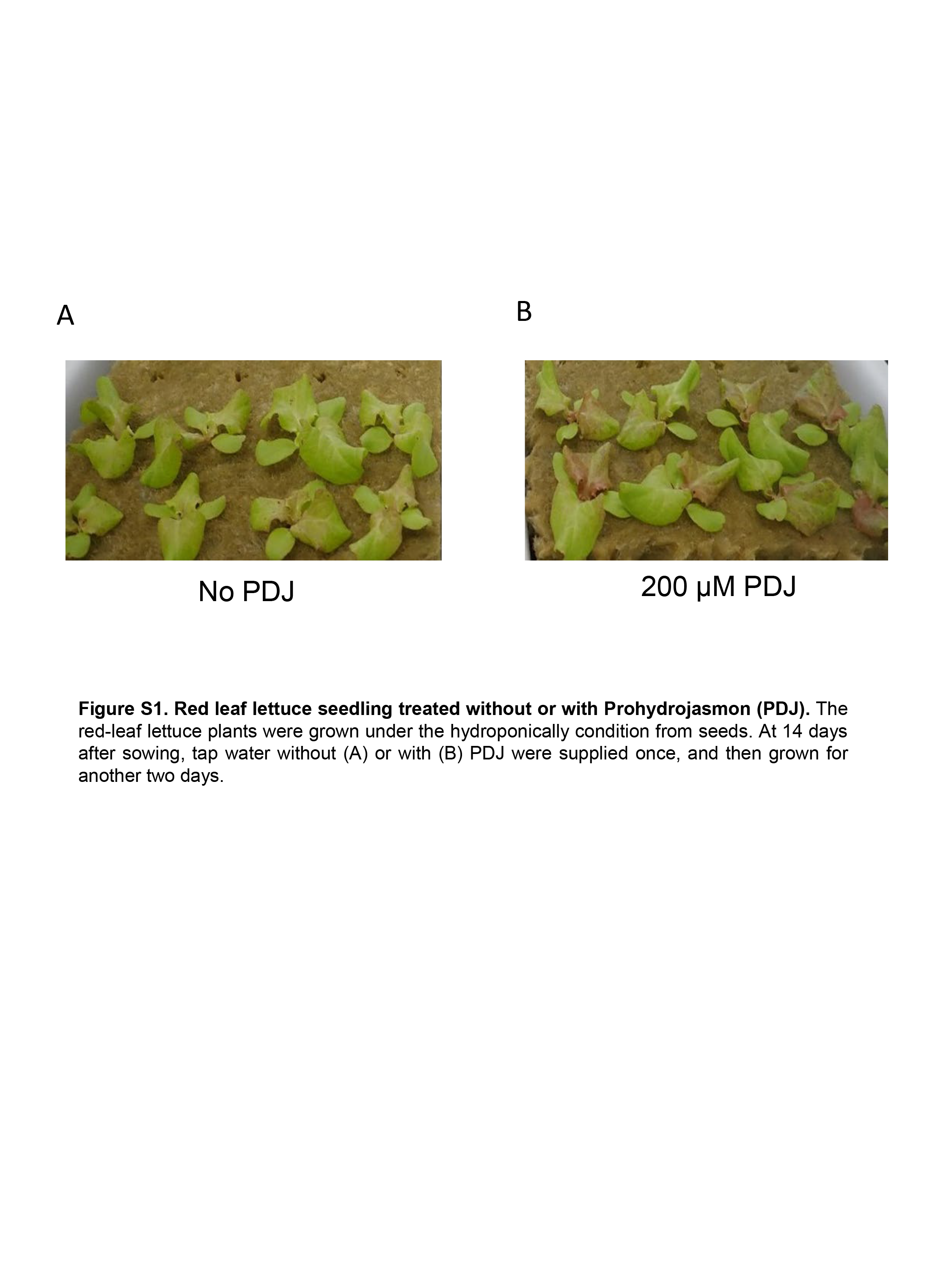

Supplement: Supplementary file 1 [file plants-10-01920-s001.zip › plants-1335827-proofed suppl/Takahashi_FigS1final.tif]

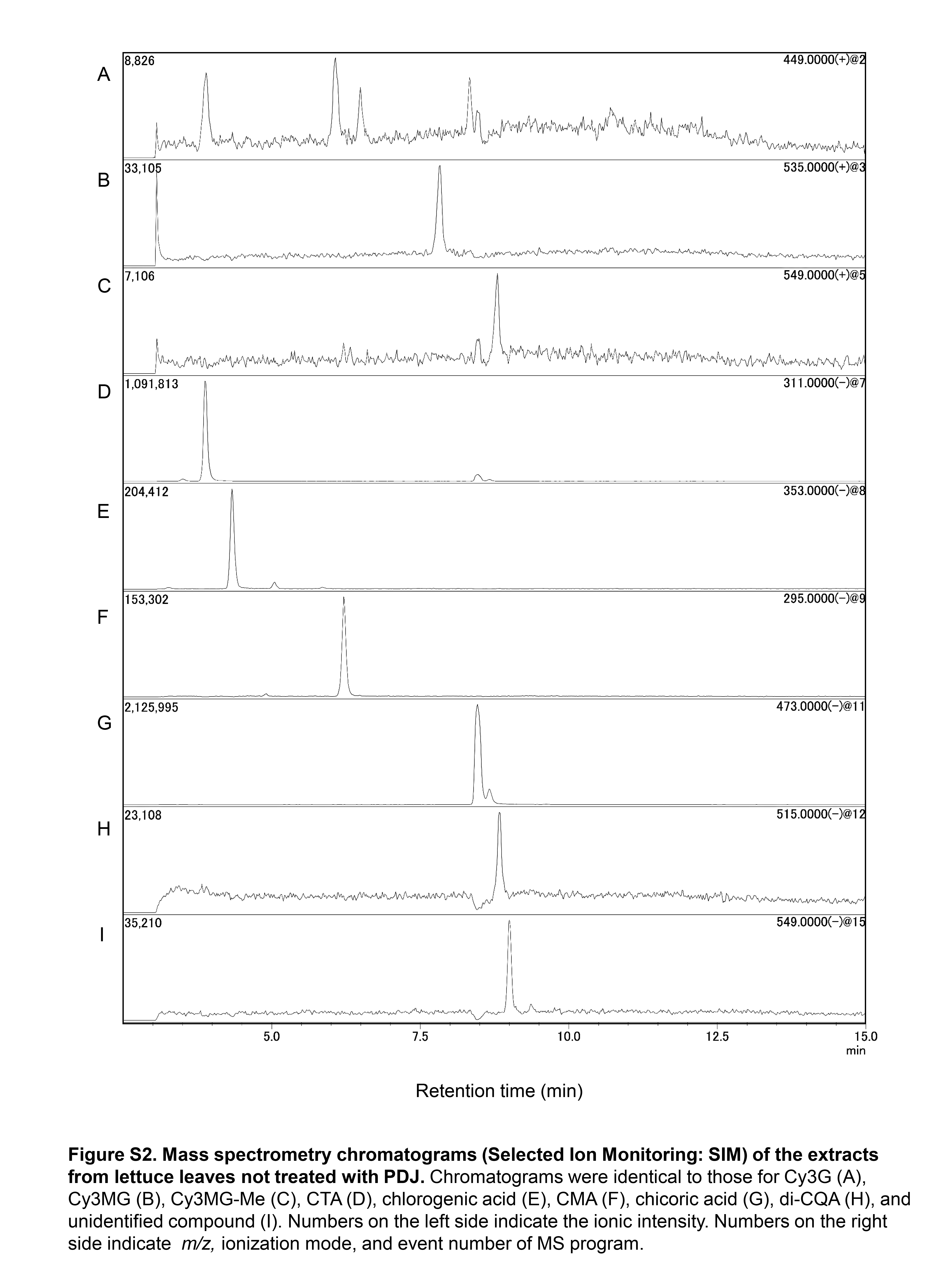

Supplement: Supplementary file 1 [file plants-10-01920-s001.zip › plants-1335827-proofed suppl/Takahashi_FigS2final.tif]

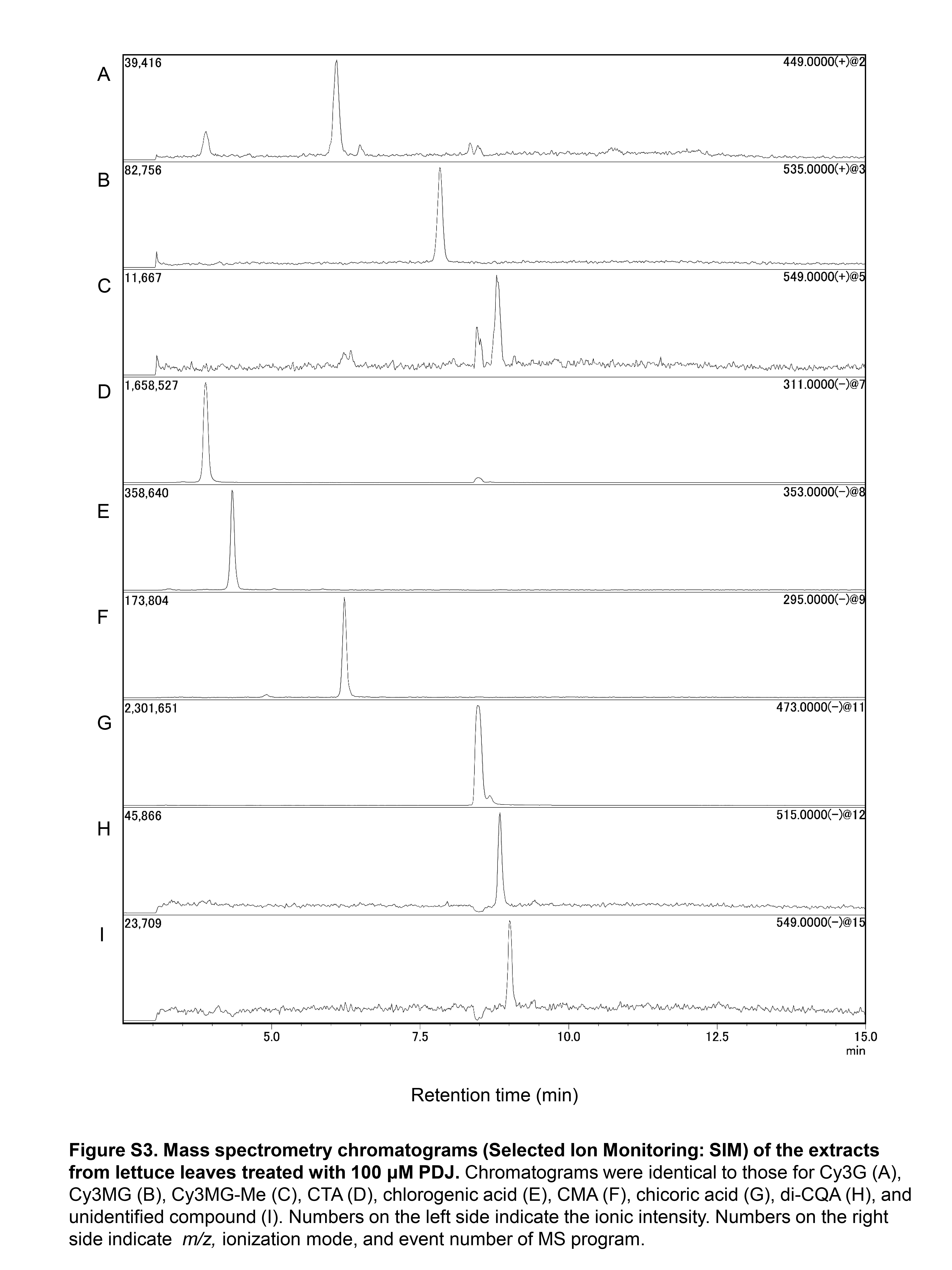

Supplement: Supplementary file 1 [file plants-10-01920-s001.zip › plants-1335827-proofed suppl/Takahashi_FigS3final.tif]

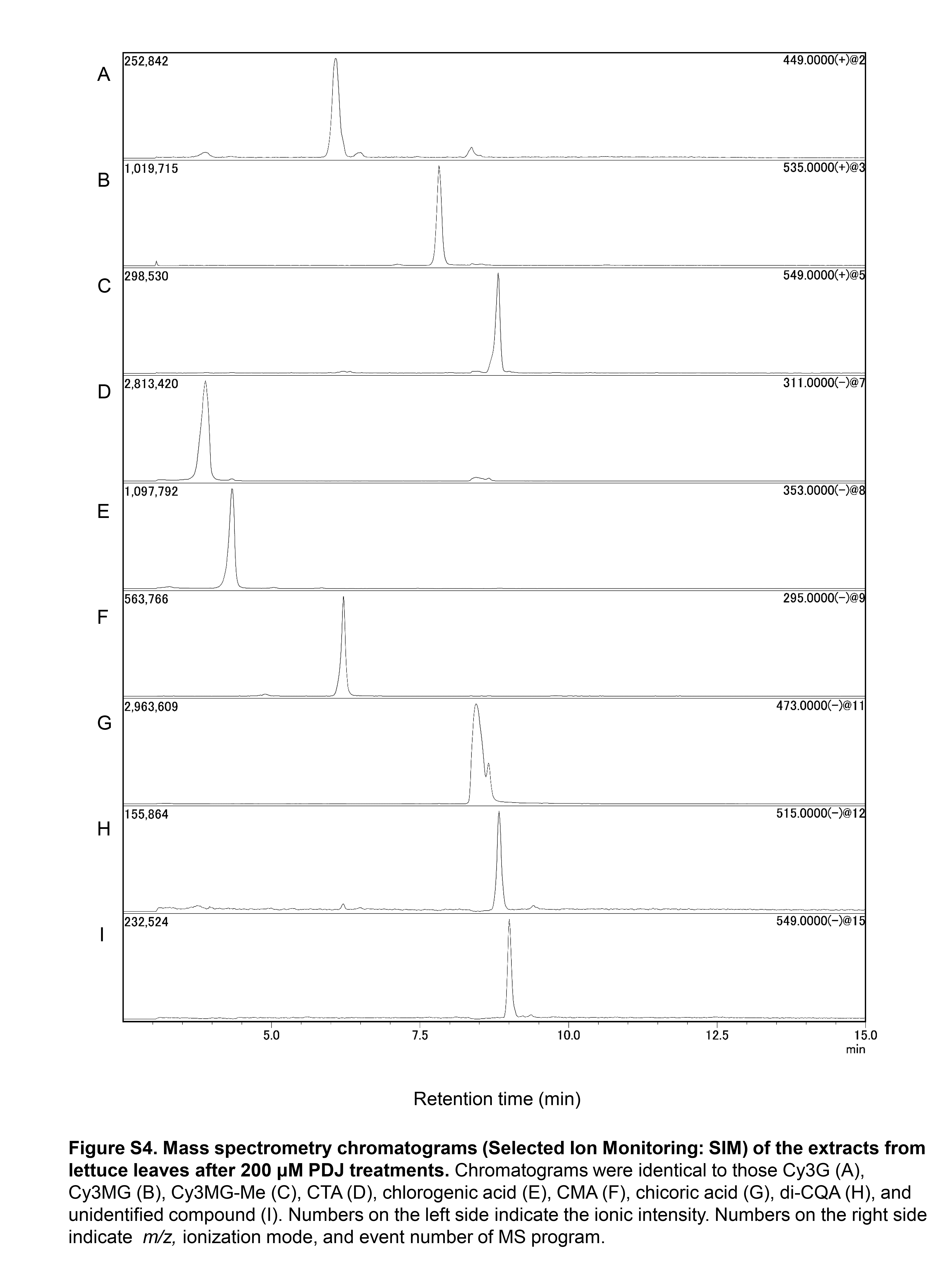

Supplement: Supplementary file 1 [file plants-10-01920-s001.zip › plants-1335827-proofed suppl/Takahashi_FigS4final.tif]

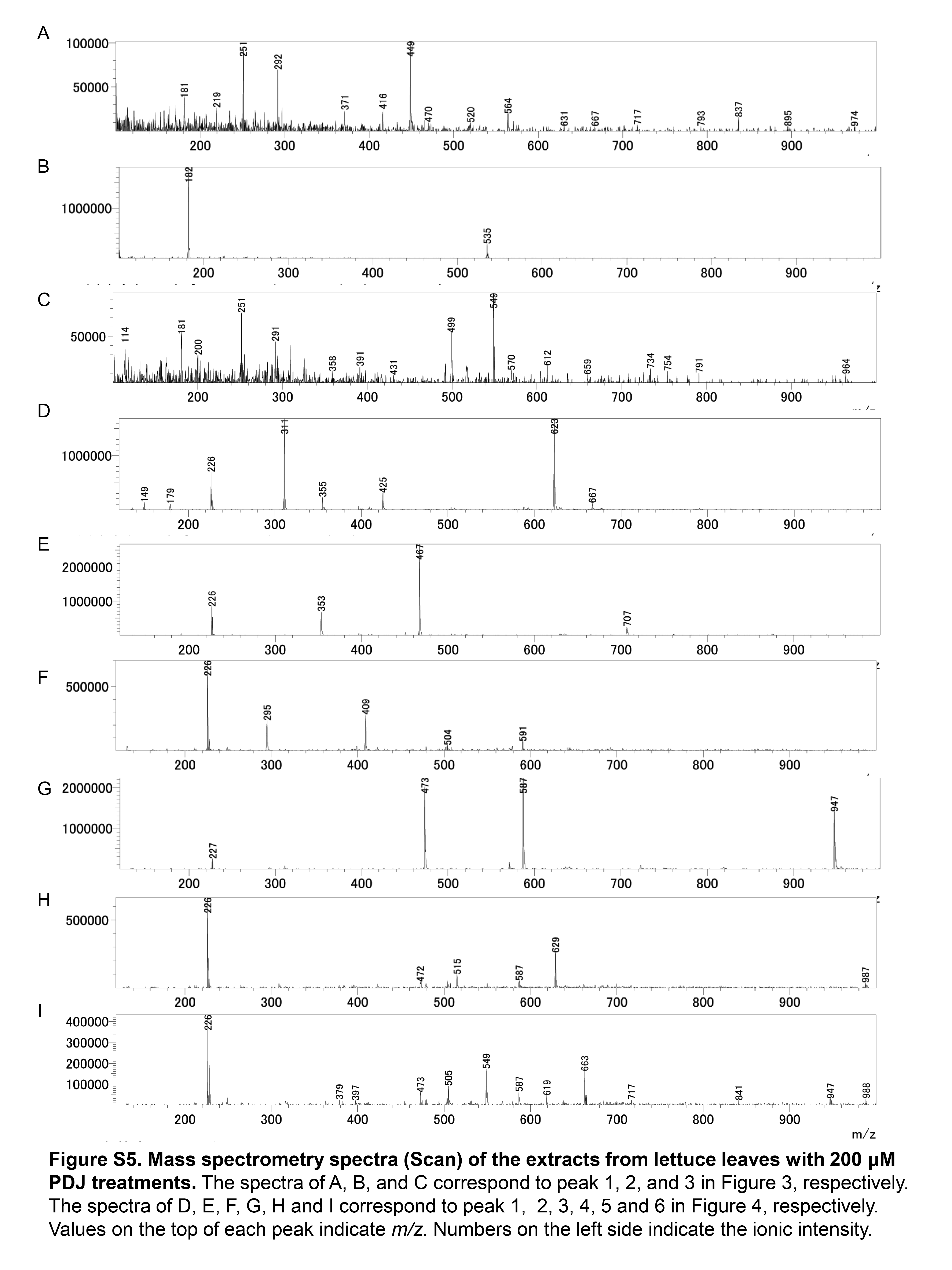

Supplement: Supplementary file 1 [file plants-10-01920-s001.zip › plants-1335827-proofed suppl/Takahashi_FigS5final.tif]

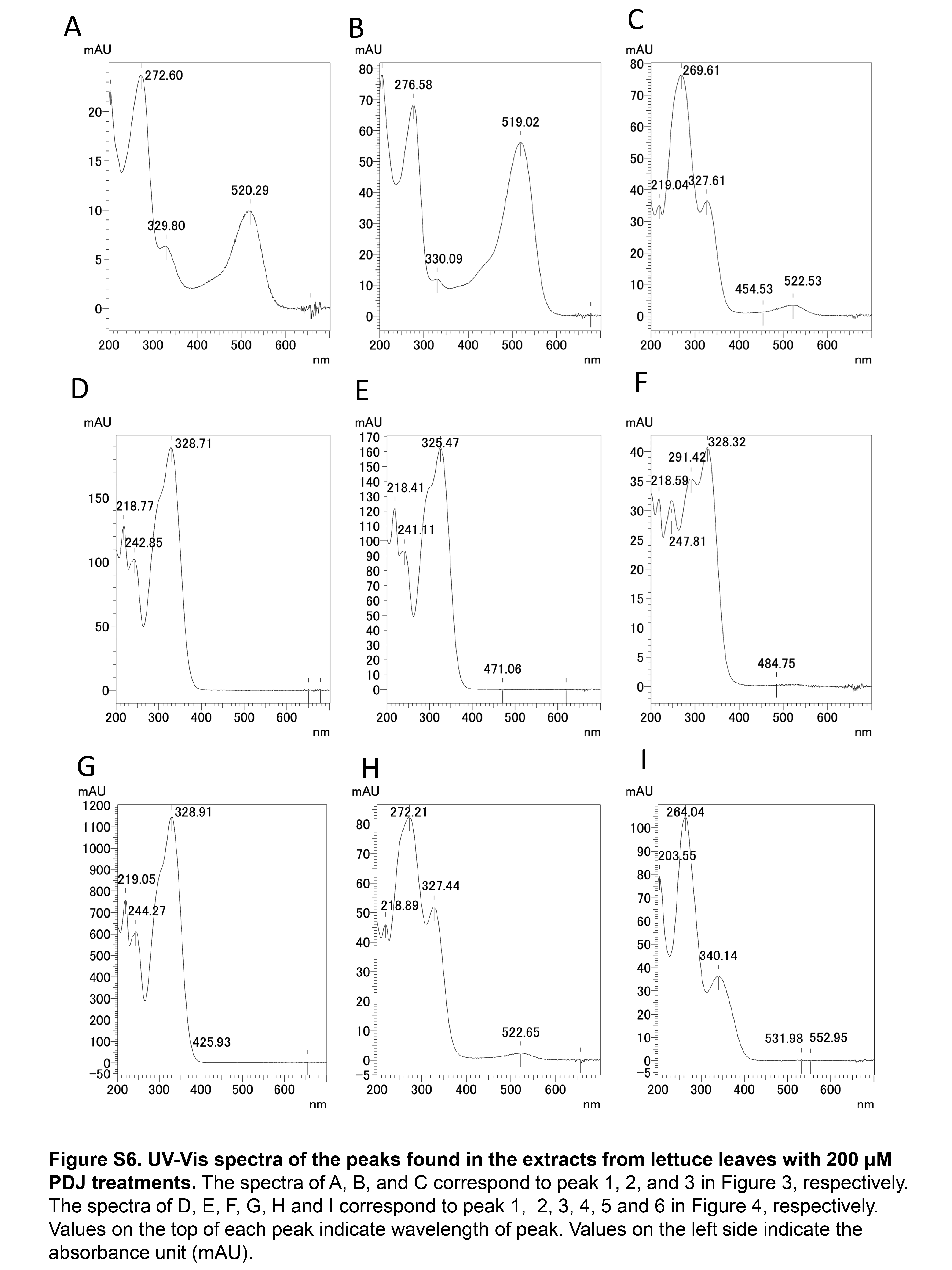

Supplement: Supplementary file 1 [file plants-10-01920-s001.zip › plants-1335827-proofed suppl/Takahashi_FigS6final.tif]

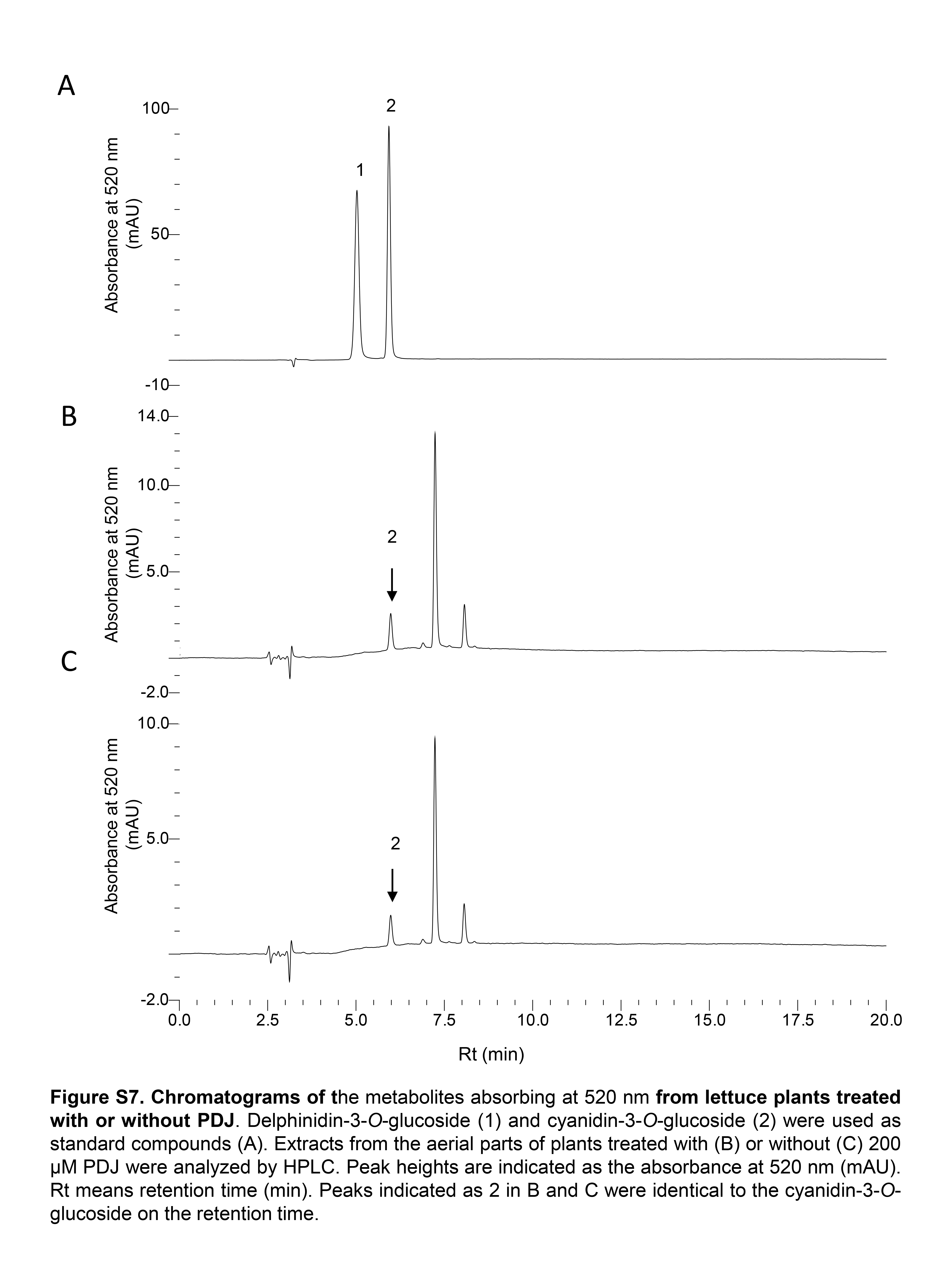

Supplement: Supplementary file 1 [file plants-10-01920-s001.zip › plants-1335827-proofed suppl/Takahashi_FigS7final.tif]

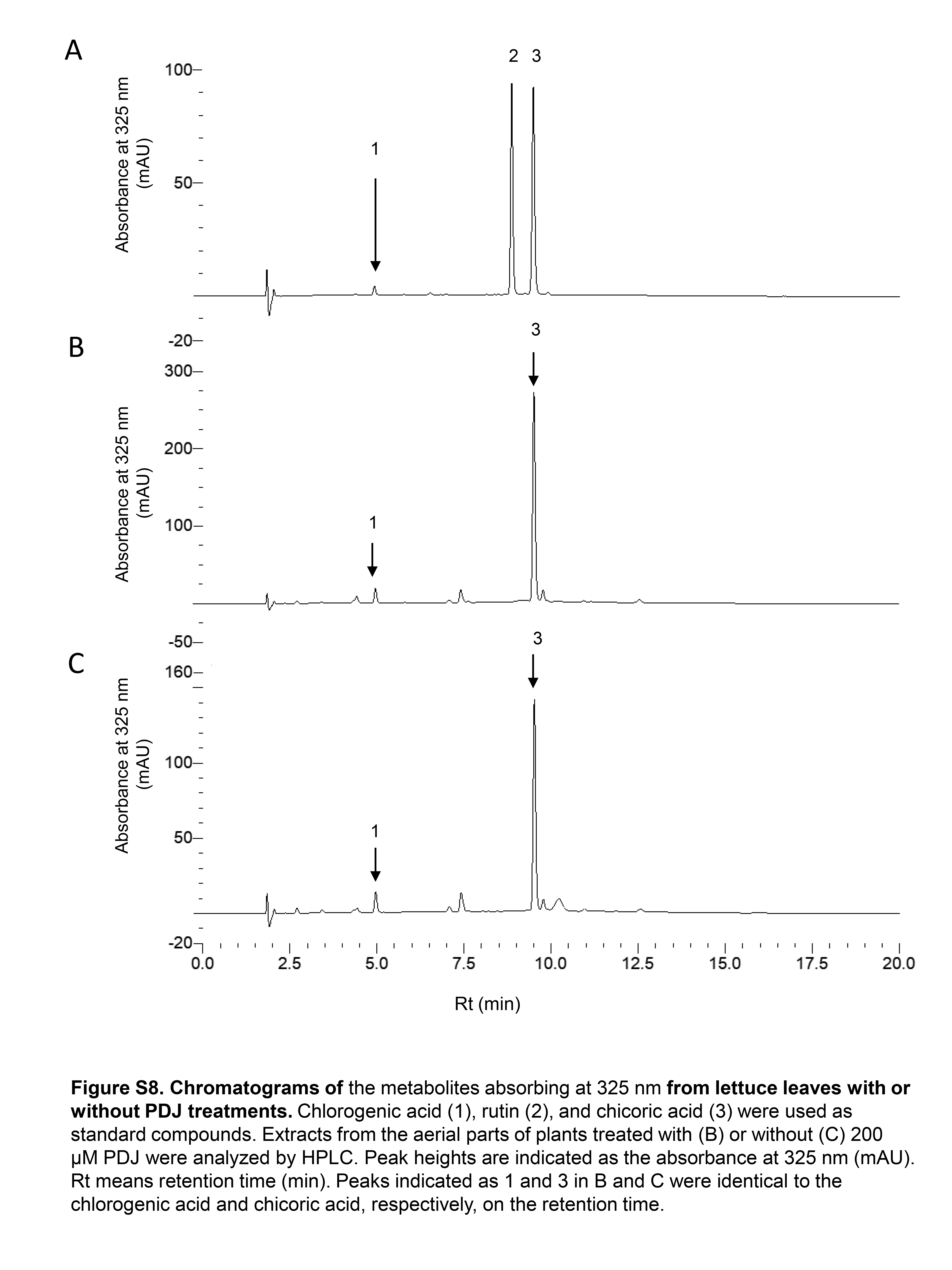

Supplement: Supplementary file 1 [file plants-10-01920-s001.zip › plants-1335827-proofed suppl/Takahashi_FigS8final.tif]

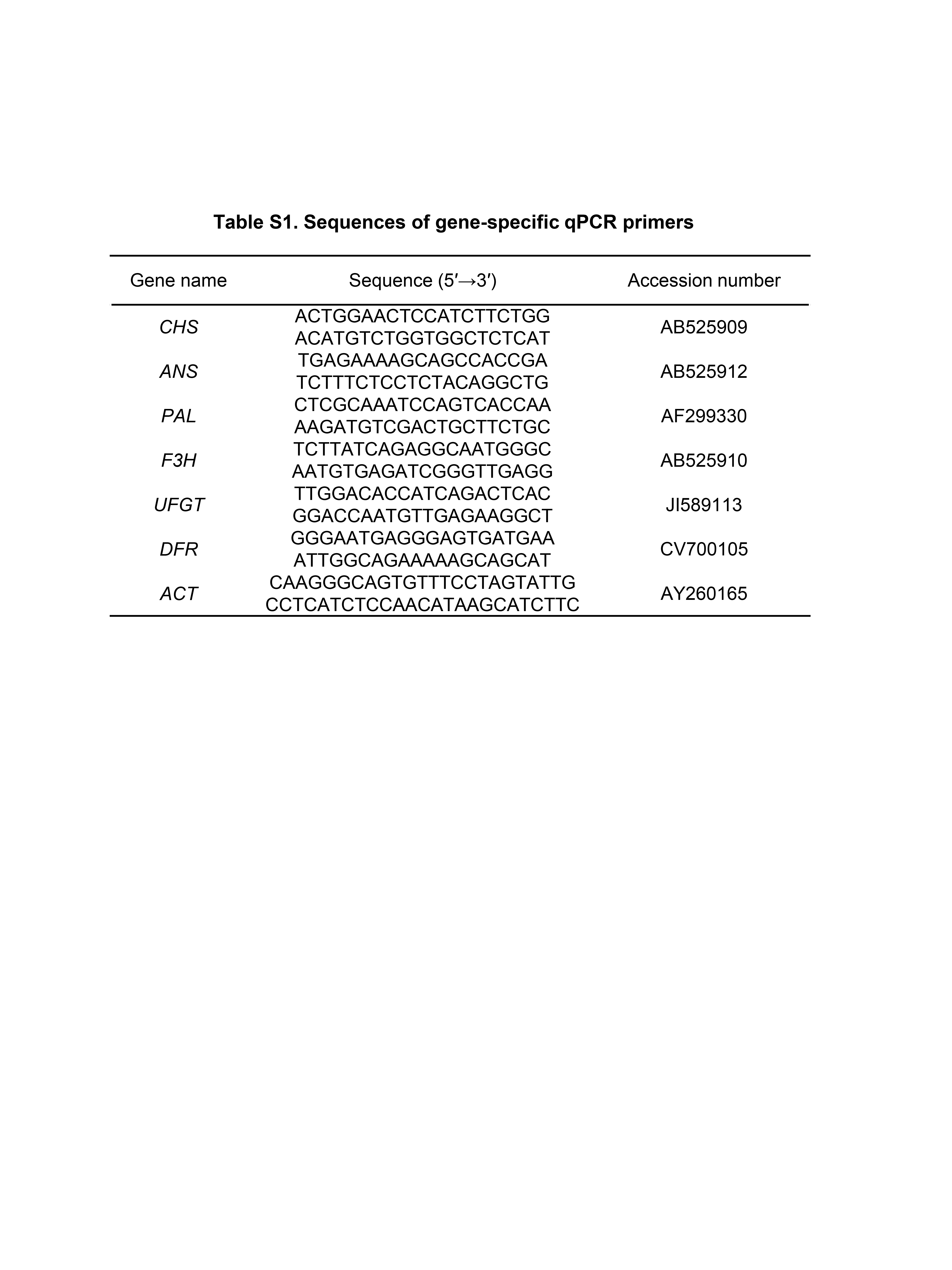

Supplement: Supplementary file 1 [file plants-10-01920-s001.zip › plants-1335827-proofed suppl/Takahashi_TableS1final.tif]
